# Autonomic imbalance captures maternal and fetal circulatory response to pre-eclampsia

**DOI:** 10.1186/s40885-016-0061-x

**Published:** 2017-02-08

**Authors:** Igor Lakhno

**Affiliations:** Kharkiv Medical Academy of Postgraduate Education, Amosova str., 58, Kharkiv, 61176 Ukraine

**Keywords:** Autonomic nervous system, Respiratory sinus arrhythmia, Pre-eclampsia

## Abstract

**Background:**

Pre-eclampsia (PE) is a gestational disease featured by hypertension, arterial systemic vasculopathy, multiple organ failure and fetal compromise.

The aim of the investigation was to determine the role of maternal respiratory sinus arrhythmia (RSA) in regulation of the fetal circulatory system in case of healthy pregnancy and in PE.

**Methods:**

The investigation of maternal and fetal HRV and fetal CTG variables in 106 patients at 34–40 weeks of gestation was performed. 30 of them had healthy pregnancy and were involved in the Group I. In Group II 44 pregnant women with mild-moderate PE were observed. 32 patients with severe PE were monitored in Group III.

**Result:**

The maternal sympathetic overactivity modulated HRV in PE by suppressing total power (TP) and parasympathetic tone. The lack of RSA was explored in preeclamptic patients. The centralization of hemodynamics was a result of the hypersympatheticotonia in severe PE. Fetal circulatory response to PE featured by an increased sympathetic tone. The modulated fetal CTG variables captured the suppression of fetal biophysical activity and the development of fetal distress in severe PE.

Strong relationship between maternal and fetal TPs, maternal and fetal RMSSDs was found in healthy pregnancy. The correlations between the maternal and fetal TPs, the maternal and fetal RMSSDs in the patients with severe PE were disturbed.

**Conclusion:**

The maternal RSA propagated its influence on the fetal autonomic nervous regulation in normal gestation. The maternal and fetal hemodynamic coupling was reduced in PE.

## Background

Pre-eclampsia (PE) is a pregnancy-associated hypertensive disorder that leads to maternal multiple organs failure and fetal compromise [1–5]. The reduced endovascular plasmatic volume is known as one of the main features of hemodynamic regimen in pre-eclamptic maternal organism. The hypovolemia is associated with an increased sympathetic activity [4, 5]. The elevated autonomic balance is an evident marker of augmented peripheral vascular tone and hypoperfusion of the end-organs [3].

The adequate trophoblastic invasion into spiral arteries is responsible for the utero-placental hemodynamics in healthy pregnancy. The lack of angiogenesis and thrombotic events are involved in placental circulatory deterioration. The insufficient invasion causes the well-known placental synthesis of pro-inflammatory substances and vasoconstrictors. Placental ischemic syndrome is an initial event in the scenario of PE [3, 4, 6]. Further endothelial dysfunction, oxidative stress and thrombophilia enhance vasoconstriction. The prediction of PE requires the investigation of different biochemical and biophysical markers [1–3].

Heart rate variability (HRV) is a famous approach to the evaluation of the cardiovascular oscillations. HRV is known as a window to the status of the human regulatory systems. HRV captures the impact of central and peripheral circuits of regulation on hemodynamics [5]. The sympathetic overactivity could be speculated as a preclinical sign of PE [3, 5].

Since maternal and fetal circulatory systems are anatomically distinct from each other the question of their interaction becomes a very relevant issue. The periods of maternal and fetal cardiac synchrony were explored [7, 8]. Maternal relaxation, physical and mental activities are known to be associated with fetal autonomic response [9–12]. Maternal respiratory sinus arrhythmia (RSA) was determined as an evident factor of maternal and fetal heart rate synchronization [7, 13].

RSA captures parasympathetic impact on the heart rate variability (HRV). This physiological phenomenon provides nonlinearity of the cardiac function and cardiorespiratory synchronization [6]. RSA is known to have a modulating impact on heart rate, cardiac output, blood pressure and peripheral vascular tone of end-organs [2, 11–13]. The decreased RSA is a sign of the cardiac failure [2, 4, 5]. Fetal RSA is one of the main factors of cardiac rhythm complexity in physiological condition. The lack of fetal parasympathetic regulation till the last weeks of healthy pregnancy was found [12]. But fetal respiratory activity is strongly associated with an increased vagal domain region of HRV [6, 14].

The speculation could be done that maternal RSA-associated hemodynamic fluctuations could penetrate through placental barrier. Therefore, these hypothesized fluctuations could be considered a possible coupling mechanism of the maternal and fetal circulatory systems. The placental vascular bed acts as an intermediary of hemodynamic oscillations. Fetal RSA is involved in its adaptive response to chronic placental insufficiency [14]. The investigation of the relationship between maternal and fetal HRV parameters could contribute to a better understanding of their role in PE.

The investigation’s aim was to determine the role of maternal RSA in regulating the fetal circulatory system in case of healthy pregnancy and in pre-eclamptic patients.

## Method

The study protocol was approved by the Bioethics Committee of the Kharkiv Medical Academy of Postgraduate Education. The eligible participants were informed about the study’s methodology, its aims, objectives, indications and eventual complications before enrollment. Patients from the department of maternal-fetal medicine were selected randomly. All the patients who met the inclusion criteria gave written informed consent to participate [15]. The inclusion criteria: diagnosed PE based on the blood pressure higher than 140/90 mmHg in two separate occasions 6 h apart, a positive proteinuria test in two mild-stream urine samples collected 4 h apart. The exclusion criteria: multiple pregnancy, eclampsia, pre-existing medical disorders like diabetes mellitus, metabolic syndrome, cardiac diseases, renal disease, thyrotoxycosis and chronic hypertension. If blood pressure was 140 to 159 mmHg systolic and 90 to 109 mmHg the patient was included in mild-moderate PE Group. Severe PE was diagnosed in case of blood pressure was higher 160 mmHg systolic and 110 mmHg diastolic or (and) thrombocytopenia, serum creatinine more than 1.1 mg/L, elevated blood concentration of liver transaminases to twice normal concentration, pulmonary oedema, cerebral or visual disturbances. The patients who had no gestational complications and medical disorders including chronic infections and tobacco smoking were enrolled in the control Group. All patients included in the study were inhabitants of Eastern Ukraine. The study was conducted from January 2013 to October 2015.

One hundred six patients at 34–40 weeks of gestation were enrolled. 30 of them had healthy pregnancy and were included into the Group I (control). In Group II, 44 pregnant women with mild-moderate PE were observed. 32 patients with severe PE were monitored in Group III.

All examined pre-eclamptic patients received antihypertensive drugs. The choice of antihypertensive agent was made according to the type of central maternal hemodynamics (CMH) determined by bio-impedance cardiography. It was estimated the values of cardiac index (CI) and total peripheral vascular resistance (TPVR). The hyperkinetic type of CMH was associated with high CI and low TPVR. The pre-eclamptic women with eukinetic type of CMH had high or normal CI and increased TPVR. And the pre-eclamptic patients with low CI and high TPVR had the hypokinetic type of CMH [3]. The pregnant women with hyperkinetic type of CMH took carvedilol 6.25–12.5 mg 2 times daily, in case of the eukinetic type – methyldopa 250–500 mg 4 times a day and in cases of the hypokinetic one – methyldopa 500 mg 4 times daily combined with nifedipine 20 mg 2 times daily.

The fetal and maternal HRV parameters were obtained with the fetal noninvasive computer electrocardiographic system “Cardiolab Baby Card” (Scientific Research Center “KhAI-Medica”, Ukraine). The Ukrainian ECG recordings were included in the Physio Net database [16]. The recording lasted for 10 min in the normal maternal sitting position. The values of total power (TP) and its spectral compounds, i.e. the very low frequency (VLF), the low frequency (LF), the high frequency (HF) and LF/HF ratio or sympatho-vagal balance, were determined. The temporal characteristics of the fetal HRV: the standard deviation of normal to normal intervals (SDNN), RMSSD, the proportion of the number of pairs of NNs differing by more than 50 ms divided by the total number of NNs (pNN50), the amplitude of mode (the most frequent value of NN interval or the highest column in the histogramm) – the number of NN intervals included in the pocket corresponding to the mode measured in percentages (%) (AMo) and the stress index – SI = AMo (%)/(2 × Mo × Var); Var = NNmax – NNmin; (SI) were calculated [17]. The fetal frequency bands of HRV were explored by David M. et al. [18]. The root mean square of successive heartbeat interval differences (RMSSD) was considered as a RSA-related parameter [14]. The level of RMSSD both in mother and fetus was investigated twice within the process of treatment in pre-eclamptic patients. The obtained fetal RR interval time series was transformed into cardiotocographic (CTG) tracing. The following CTG parameters were determined: short term variation (STV), long term variation (LTV) and the number of accelerations and decelerations.

The results thus obtained were analyzed with an ANOVA test to compare data between groups. The significance was set at *p*-value <0.05. For the statistical analysis of relationship between X and Y, the correlations coefficients were estimated with Spearman’s test. Microsoft Office 2010 Excel software was used for statistical analysis (Washington, USA).

## Result

The mean age values were 26.5 ± 4.1; 25.8 ± 7.2 and 25.4 ± 6.3 years in Group I, Group II and Group III respectively. The mean values of the gestational age were 37.1 ± 3.6; 36.9 ± 2.5 and 36.7 ± 1.8 weeks in Group I, Group II and Group III respectively. The body mass index values in the same groups were 24.9 ± 5.1; 28.5 ± 7.8 and 29.6 ± 8.3. So, the mean values of body mass index in PE were significantly higher than in healthy pregnancy Group (*p* < 0.05).

The study of CMH types revealed an increased both CI and TPVR mean values in mild-moderate PE Group (Table 1). The mean value of CI was low and the mean value of TPVR was elevated in severe PE. Therefore, an increase in pre- and afterload on maternal heart in Group III was found. The hyperkinetic type of CMH was found in 86.4% and the eukinetic one in 13.6% of the patients in Group II. 59.4% of women in Group III had the hypokinetic type of CMH and 40.6% of severe pre-eclamptic patients had a eukinetic pattern of CMH. Hyperdynamic circulation was typical for mild-moderate PE and hypodynamic one was found in severe PE. Therefore, the patients of Group III had a centralization of blood flow.

**Table 1:** The parameters of bioimpedance cardiography in the study population

Index, units of measure	Group I	Group II	Group III
CI, L/min/m^2^	3.6 ± 0.8	3.9 ± 1.2*	2.2 ± 1.1*⁄^†^
TPVR, dyn · s/cm^5^	1214.5 ± 128.2	1371.0 ± 203.4*	2460.2 ± 318.6*⁄^†^

*the differences were statistically significant compared to the control group (*p* < 0,05)

^†^the differences were statistically significant compared to the group II (*p* < 0,05)

The obtained data showed a suppressed autonomic tone in pre-eclamptic patients (Table 2). The most considerable power was determined in the maternal VLF domain region in all study groups. It was linked with a relative predominance of the hypothalamic-pituitary-adrenal axis domain region among all spectral components of HRV in sitting position in a state of rest. The maternal HRV in PE demonstrated an augmented activity of the central sympathetic circuit. This peculiarity was associated with the relative increase of AMo, SI and LF. The mean sympatho-vagal balance (LF-to-HF ratio) values were 0.9 ± 0.3, 2.2 ± 0.6 and 4.5 ± 1.1 respectively in Group I, Group II and Group III. This gradual growth of sympatho-vagal balance was associated with the progredient severity of PE. The mean values of short-term parameters: the RMSSD, the pNN5O and the HF were lower in Group II and Group III. The lack of parasympathetic regulation captured a decreased impact of RSA on maternal hemodynamics.

**Table 2:** Maternal HRV parameters in the study population

Index	Group I	Group II	Group III
SDNN, ms	119. 8 ± 14.1	102.5 ± 9.0*	82.6 ± 10.4*⁄^†^
RMSSD, ms	41.6 ± 8.5	22.7 ± 6.2*	16.3 ± 4.8*⁄^†^
pNN5O, %	12.8 ± 3.2	6.5 ± 1.9*	1.8 ± 0.6*⁄^†^
AMo, %	34.6 ± 5.1	50.4 ± 11.3*	65.4 ± 12.1*/^†^
SI, c.u.	115.2 ± 16.8	403.9 ± 34.5*	1362.6 ± 243.4*⁄^†^
TP, ms^2^	3084.6 ± 565.7	1568.2 ± 347.2*	825.6 ± 117.9*⁄^†^
VLF, ms^2^	2361.2 ± 485.3	1130.8 ± 181.4*	541.6 ± 85.2*⁄^†^
LF, ms^2^	349.5 ± 42.6	310.3 ± 51.6*	231.9 ± 52.4*⁄^†^
HF, ms^2^	375.4 ± 56.1	128.6 ± 31.4*	53.1 ± 13.6*⁄^†^
LF/HF	0.9 ± 0.3	2.2 ± 0.6*	4.5 ± 1.1*⁄^†^

*Abbreviations*: *SDNN* the standard deviation of normal to normal intervals, *RMSSD* the root mean square of successive heartbeat interval differences, *pNN50* the proportion of NN pairs differing by more than 50 ms divided by total number of NNs, *AMo* the mode amplitude (the most frequent value of NN interval or the highest column in the histogramm) – the number of NN intervals included into the pocket corresponding to the mode measured in percentages (%) (AMo), *SI* the stress index SI = AMo (%)/(2 × Mo × Var); Var = NNmax – NNmin, *TP* the total power, *VLF* the very low frequency, *LF* the low frequency, *HF* the high frequency

*the differences were statistically significant compared to Group I (*p* < 0.05)

^†^the differences were statistically significant compared to Group II (*p* < 0.05)

The fetal HRV parameters demonstrated a suppressed autonomic nervous regulation with an abnormal relative elevation of the sympathetic domain region values in PE (Table 3). The values of fetal SDNN and TP were lower in Group II and Group III. However, the share of LF grew relatively in the total spectra of fetal HRV in the pre-eclamptic patients. The considerable growth of AMo and SI values was found. The decrease of RMSSD, pNN5O and HF in the patients with PE was determined in Group II and Group III. So, the reduced fetal vagal activity was found in PE. Fetal RSA according to RMSSD value was decreased too. Therefore, sympathetic overactivity had a modulating impact on RSA by suppressing the power of the parasympathetic domain region. The revealed CTG indices demonstrated the decreased fetal heart rate reactivity in patients with PE. The values of STV, LTV and number of accelerations were significantly lower in patients of Group II and Group III. The number of decelerations was increased in pre-eclamptic Groups comparatively to healthy pregnancy. The modulated fetal CTG variables captured the suppression of fetal biophysical activity and the development of fetal distress in severe PE.

**Table 3:** Fetal HRV parameters and CTG indices in the study population

Index	Group I	Group II	Group III
SDNN, ms	45.8 ± 13.1	29.4 ± 8.3*	10.2 ± 4.5*⁄^†^
RMSSD, ms	22.4 ± 3.4	14.2 ± 2.6*	8.1 ± 0.8*⁄^†^
pNN5O, %	4.2 ± 1.1	2.0 ± 0.4*	1.1 ± 0.3*⁄^†^
AMo, %	39.6 ± 14.1	50.2 ± 11.6*	65.9 ± 13.4*/^†^
SI, c.u.	169.3 ± 42.7	496.1 ± 65.8*	1467.3 ± 405.8*⁄^†^
TP, ms^2^	1513.6 ± 329.1	896.2 ± 163.5*	424.9 ± 93.7*⁄^†^
VLF, ms^2^	1252.8 ± 248.3	692.8 ± 91.3*	251.8 ± 44.2*⁄^†^
LF, ms^2^	184.3 ± 26.5	151.9 ± 34.1*	135.0 ± 19.6*⁄^†^
HF, ms^2^	77.6 ± 9.4	53.6 ± 8.2*	38.9 ± 10.4*⁄^†^
STV, ms	7.5 ± 2.8	6.0 ± 2.2*	4.1 ± 1.6*⁄^†^
LTV, ms	21.9 ± 7.1	16.1 ± 5.8*	12.6 ± 4.3*⁄^†^
Number of accelerations	4.2 ± 1.8	2.7 ± 1.3*	1.2 ± 0.4*⁄^†^
Number of decelerations	0.3 ± 0.1	1.5 ± 0.4*	2.8 ± 0.9*⁄^†^

*Abbreviations*: *SDNN* the standard deviation of normal to normal intervals, *RMSSD* the root mean square of successive heartbeat interval differences, *pNN50* the proportion of NN pairs differing by more than 50 ms divided by the total number of NNs, *AMo* the mode amplitude (the most frequent value of NN interval or the highest column in the histogramm) – the number of NN intervals included in the pocket corresponding to mode measured in percentages (%), *SI* the stress index SI = AMo (%)/(2 × Mo × Var); Var = NNmax – NNmin, *TP* the total power, *VLF* the very low frequency, *LF* the low frequency, *HF* the high frequency

*the differences were statistically significant compared to Group I (*p* < 0.05)

^†^the differences were statistically significant compared to Group II (*p* < 0.05)

The values of maternal and fetal RMSSDs were not changed significantly in the process of treatment. The interval between RMSSDs assessments was different in the study Groups. The mean interval values were 7.2 ± 1.5 days and 4.1 ± 1.8 days in Group II and Group III respectively. Such variation was associated with the necessity of the preterm pregnancy termination in patients with severe PE.

It was found a weak relationship between sympatho-vagal balance and CI (*R* = −0.30; *p* < 0.05), sympatho-vagal balance and TPVR (*R* = 0.32; *p* < 0.05) in healthy pregnancy Group (Table 4). In mild-moderate PE Group the strength of correlation in the pairs: sympatho-vagal balance versus CI (*R* = −0.34; *p* < 0.05) and sympatho-vagal balance versus TPVR (*R* = 0.38; *p* < 0.05) was found almost on the previous level. The strength of correlation was strong in the same pairs: sympatho-vagal balance versus CI (*R* = −0.63; *p* < 0.05) and sympatho-vagal balance versus TPVR (*R* = 0.70; *p* < 0.05) in severe PE Group.

**Table 4:** Statistically significant (*p* < 0.05) Spearman’s correlations between the maternal sympatho-vagal balance and CI, maternal sympatho-vagal balance and TPVR in the study population

Pairs of parameters (X versus Y)	Group I	Group II	Group III
Sympatho-vagal balance versus CI	*R* = −0,30	*R* = −0,34	*R* = −0,63
Sympatho-vagal balance versus TPVR	*R* = 0,32	*R* = 0,38	*R* = 0,70

The investigation of statistically significant correlations between the maternal and fetal RMSSDs and TP worked out certain regularities (Table 5). The most considerable positive correlation was determined in the healthy pregnancy Group between the maternal and fetal TP (*R* = 0.64; *p* < 0.05), the maternal RMSSD and the fetal RMSSD (*R* = 0.51; *p* < 0.05). The positive weak correlation between the maternal and fetal TPs (*R* = 0.34; *p* < 0.05) was determined in Group II. The positive weak correlation between the maternal and fetal RMSSDs in women with mild-moderate PE (*R* = 0.36; *p* < 0.05) was also found. No considerable relationship was worked out between the maternal and fetal TPs (*R* = 0.20; *p* < 0.05), the maternal and fetal RMSSDs (*R* = 0.18; *p* < 0.05) in the patients with severe PE.

**Table 5:** Statistically significant (*p* < 0.05) Spearman’s correlations between the maternal and fetal TPs, the maternal and fetal RMSSDs in the study population

Pairs of parameters (X versus Y)	Group I	Group II	Group III
Maternal TP versus fetal TP	*R* = 0.64	*R* = 0.36	*R* = 0.20
Maternal RMSSD vs fetal RMSSD	*R* = 0.51	*R* = 0.32	*R* = 0.18

## Discussion

The obtained data supported the well-known hyperdynamic model of the mild-moderate PE. The persistent gestational hypervolemia with an augmented periferal vascular tone provided an increased cardiac output. Further hemodynamic crossover to hypovolemia and low cardiac output manifested hypodynamic circulation in severe PE [3]. The increased sympathetic activity in the mild-moderate PE could be considered a compensatory reaction. Such circulatory response captured an adaptive support of the maternal organs perfusion. The severe PE was associated with maximal sympathetic tone amplification. High peripheral vascular resistance and hypovolemia caused hypoperfusion of the end-organs in severe pre-eclamptic patients. Therefore, severe PE destroyed gestational adaptive autonomic response and contributed to the development of cardiac failure.

A parasympathetic regulation was proved more dominant than the sympathetic one in reproductive-aged women. The parasympathetic division of autonomic nervous system was found to be involved in the gestation regulatory resetting and provided an increased level of ergo-, trophotropic reactions and physiological fluid retension [4]. Therefore, the vagal regulation demonstrated a considerable ability to protect the gravidity in the first half of healthy pregnancy [2, 5]. The current study worked out an increased power of the sympathetic domain region in the total HRV spectra of pre-eclamptic women. It was previously determined that the sympathetic tone was more than 3 times higher in PE than in normotensive pregnant women [5]. In addition to the sympathetic overactivity, a decreased parasympathetic tone was also found in PE. The RSA is known to have a strong relationship with the parasympathetic division of autonomic nervous regulation [14]. Therefore, the suppression of parasympathetic regulation reduced the role of RSA in the total level of maternal HRV in PE. So, the RSA had a slight influence on the maternal hemodynamics in PE.

The maximal increase of the sympatho-vagal balance was a sign of the vasoconstriction and the hypoperfusion. Previously the placental bed and renal vessels were determined as the highest vascular resistance areas in PE [3, 5]. The hypothesis that elevated intraabdominal pressure causes renal and hepatic compartmentalization and increases sympatho-vagal balance and decreases RMSSD could be supported [4]. Therefore, the additional pressure could contribute to aortocaval compression and decreases the return of venous blood to maternal heart in PE. The emphasis on the preload requires sympathetic overactivity [3]. The above-mentioned autonomic modulation could suppress RSA. The abnormally elevated value of sympatho-vagal balance is known to be associated with abdominal compartmentalization in severe PE [4].

Even reasonable usage of antihypertensive drugs failed to decrease the autonomic balance in PE. The level of RMSSDs in mother and fetus persists to stay on the same in the process of treatment. Therefore, severe PE destroyed the basis for the pregnancy vagal-mediated mechanism of fluid retention and vasodilation. That is why the only efficient treatment of the severe pre-eclamptic patients is a pregnancy termination. Possibly the postpartum decrease of the intraabdominal pressure could contribute to the sympatho-vagal balance reestablishment.

The fetal HRV demonstrated a pathological pattern of the autonomic nervous regulation in PE. A gradual reduction of fetal sympatho-vagal balance in the last trimester of healthy pregnancy was previously showed [18–21]. The increased parasympathetic regulation was a sign of the fetal maturation in the late gestation. The revealed fetal hypersympatheticotonia and the lack of vagal activity were characteristic of PE [17, 20–22]. The non-reactive pattern of fetal CTG tracing was typical for pre-eclamptic women. Thus, PE caused fetal distress and changed its autonomic response. It was found in the previous study that fetal RSA in the growth restricted fetuses was on the same level with fetal RSA in healthy pregnancy [14]. This peculiarity was regarded as an adaptive response in case of normal utero-placental hemodynamics. The decreased fetal RSA in pre-eclamptic patients was explored in this study. PE had a negative impact on fetal RSA.

Several investigations described the fetal and maternal cardiac rhythm synchronization [7, 8, 13]. The relationship between maternal and fetal RMSSDs in healthy pregnancy was found. The maternal parasympathetic regulation and RSA had a protective impact on the circulatory response of the mother and fetus in PE. The increased maternal autonomic balance supported perfusion of the end organs and coupling with fetal hemodynamics in mild-moderate PE while vagal-mediated reactions were safe. Hypokinetic type of CMH in severe PE was associated with the reduction of RSA. The deteriorated placental perfusion and the decreased RSA-associated hemodynamic fluctuations were due to hemodynamic failure. Therefore, the fetal cardiovascular system was absolutely separated from the maternal organism and lost the control in severe PE (confirmed with the absent relationship between maternal and fetal TPs and RMSSDs). For this reason, the loss of fetal and maternal hemodynamic coupling could be considered a presumable pathogenetic mechanism of the fetal distress in PE. This hypothesis, though, requires further investigations. The speculation could be done that the testing for maternal sympatho-vagal balance, fetal and maternal RMSSD is possible to be of use in future as biophysical markers of PE. The screening for PE in III trimester is necessary for timely preterm pregnancy termination [23]. Such active management is a promising approach to the improvement of perinatal and maternal outcomes.

## Conclusion

The maternal RSA propagated its influence on the fetal autonomic nervous regulation in healthy pregnancy. The maternal and fetal hemodynamic coupling was reduced in PE. The hypokinetic type of CMH in severe PE decreased the RSA-mediated hemodynamic fluctuations. It was associated with fetal TP and RMSSD considerable reduction.

## Abbreviations

AMo: The mode amplitude (the most frequent value of NN interval or the highest column in the histogramm), the number of NN intervals included into the pocket corresponding to the mode measured in percentages (%) (AMo)
CI: Cardiac index
CMH: Central maternal hemodynamics
HF: The high frequency
HRV: Heart rate variability
LF: The low frequency
PE: Pre-eclampsia
pNN50: The proportion of NN pairs differing by more than 50 ms divided by total number of NNs
RMSSD: The root mean square of successive heartbeat interval differences
RSA: Respiratory sinus arrhythmia
SDNN: The standard deviation of normal to normal intervals
SI: The stress index SI = AMo (%)/(2 × Mo × Var) Var = NNmax – NNmin
TP: The total power
TPVR: Total peripheral vascular resistance
VLF: The very low frequency
